# Evolution and diversity of transposable elements in fish genomes

**DOI:** 10.1038/s41598-019-51888-1

**Published:** 2019-10-28

**Authors:** Feng Shao, Minjin Han, Zuogang Peng

**Affiliations:** 1 Key Laboratory of Freshwater Fish Reproduction and Development (Ministry of Education), Southwest University School of Life Sciences, Chongqing, 400715 China; 2 State Key Laboratory of Silkworm Genome Biology, Key Laboratory for Sericulture Functional Genomics and Biotechnology of Agricultural Ministry, Southwest University, Chongqing, 400715 China

**Keywords:** Evolutionary genetics, Mobile elements

## Abstract

Transposable elements (TEs) are genomic sequences that can move, multiply, and often form sizable fractions of vertebrate genomes. Fish belong to a unique group of vertebrates, since their karyotypes and genome sizes are more diverse and complex, with probably higher diversity and evolution specificity of TE. To investigate the characteristics of fish TEs, we compared the mobilomes of 39 species, and observed significant variation of TE content in fish (from 5% in pufferfish to 56% in zebrafish), along with a positive correlation between fish genome size and TE content. In different classification hierarchies, retrotransposons (class), long terminal repeat (order), as well as *Helitron*, *Maverick*, *Kolobok*, *CMC*, *DIRS*, *P*, *I*, *L1*, *L2*, and *5S* (superfamily) were all positively correlated with fish genome size. Consistent with previous studies, our data suggested fish genomes to not always be dominated by DNA transposons; long interspersed nuclear elements are also prominent in many species. This study suggests *CR1* distribution in fish genomes to be obviously regular, and provides new clues concerning important events in vertebrate evolution. Altogether, our results highlight the importance of TEs in the structure and evolution of fish genomes and suggest fish species diversity to parallel transposon content diversification.

## Introduction

In addition to functional genes, the genome contains numerous scattered repeats known as transposable elements (TEs). Published first in the 1950s by Barbara McClintock^1^, TEs can ‘jump’ from one chromosomal site to another. Transposable elements are either retrotransposons or DNA transposons based on whether they use RNA or DNA for mobilization, respectively. Below this broader categorization, TEs are grouped into five classes according to enzymology, structure, and sequences: long terminal repeat (LTR) retrotransposons, non-LTR retrotransposons (e.g., long and short interspersed nuclear elements, or LINEs and SINEs), cut-and-paste DNA transposons, rolling-circle DNA transposons (*Helitrons*), and self-synthesizing DNA transposons (*Polintons*)^2^.

Initially, TEs did not attract much scientific attention because they were erroneously treated as “junk DNA.” However, they are now recognized as critical functional and evolutionary components of the genome^3,4^, involved in processes such as speciation^5^, sex determination^6–9^, new gene creation^10,11^, and chromosome rearrangement^12^. Overall, TEs appear to drive genetic diversification and provide genetic material during genome evolution^13,14^. Thus, evaluating TEs is essential to the investigation of genome evolution dynamics.

Fishes are the oldest and largest group of vertebrates. Their long evolutionary history includes multiple rounds of whole genome duplication and re-diploidization events that increased their genetic complexity, so, fishes have more complex karyotypes and more diverse genome sizes than any other vertebrate taxon^15^. Notably, this taxonomic diversity is paralleled by extensive variation in genetic and phenotypic characteristics, as well as by the presence of TEs. It would be important to stress that cytological haploid genome size of fish ranges very widely (0.35–133 Gb)^16^, C value paradox arises from widely different C-values like these. Genome size has been implicated in several phenotypic traits, including cell size^17,18^ and metabolic rate^19,20^. Thus, disentangling the forces and mechanisms that regulate genome size is critical for a better understanding of piscine molecular evolution. Unfortunately, data concerning fish TEs are limited, and a detailed systematic comparative study is yet to be attempted. Rapid advancements in sequencing technology, however, has resulted in the publication of several fish genomes, providing a means to comprehensively study fish TEs. In a previous study, we created a fish-specific TE database (FishTEDB)^21^ to facilitate research on TE function and evolution in fish genomes, but we have not applied the database for systematic evaluation of TE diversity.

Therefore, this study expanded on the original FishTEDB through the addition of TE data from nine fish species. The updated database contains 39 species genomes, including 35 from Actinopterygii (14 orders), 1 from Chondrichthyes, 1 from Sarcopterygii, 1 from Agnatha, and 1 from Chordata. We used these data to evaluate correlations between TE content and genome size across different classification hierarchies, with the aim of exploring how different TE categories contribute to genome-size evolution. Furthermore, based on TE diversity, we attempted to clarify TE effects on fish evolution and explain TE specificity in fishes that occupy key positions in the evolutionary tree.

## Results

### TE content diversity and its contribution to fish genome size

**Figure 1:**
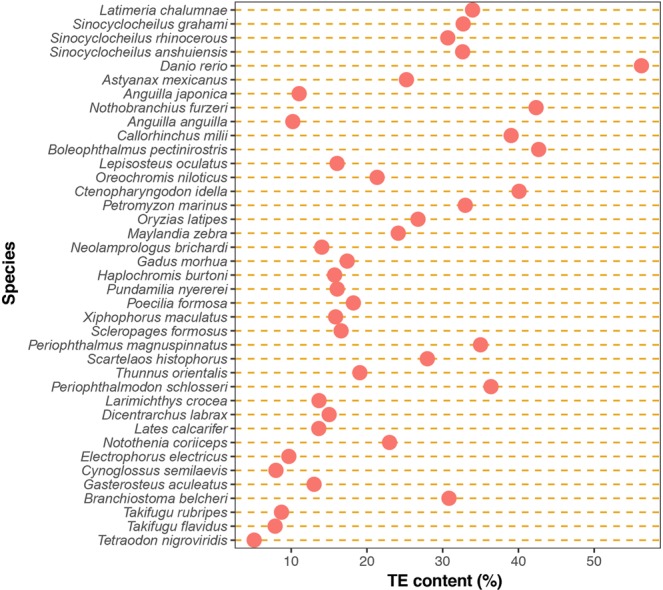
Total TE content of all species analysed in this study, sorted by genome size.

**Figure 2:**
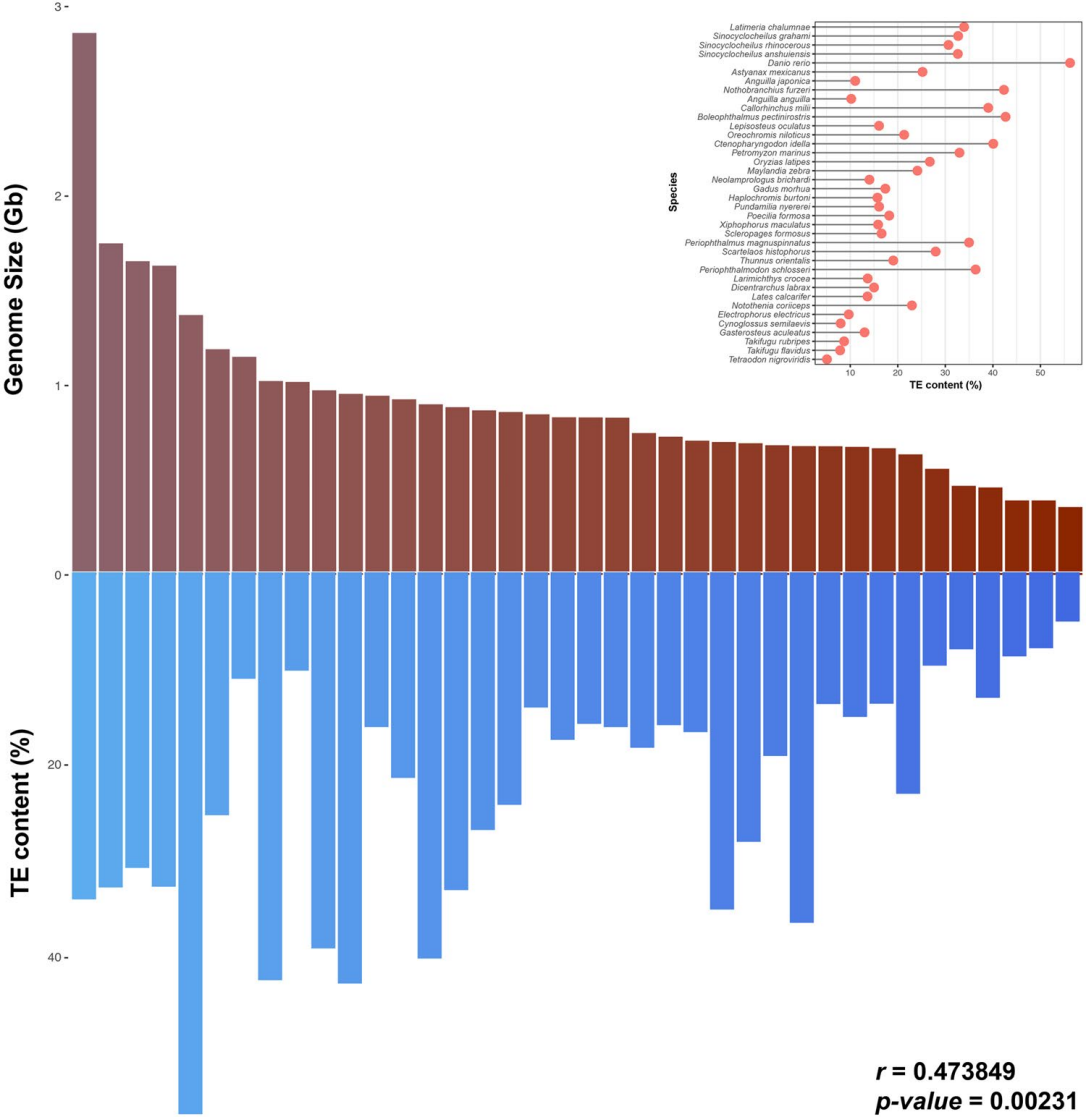
Correlation between genome size and TE content of fish. The histogram above the graph (in red) shows the distribution of genome size (unit, 1000 megabases) while that below the graph (in blue) shows TE distribution. Correlation analysis was performed by the Pearson method using the R program.

In global level (Fig. 1, Supplementary Table S2), we found TE content to be variable, ranging from 5% in pufferfish to 56% in zebrafish, and was positively correlated (Pearson correlation r = 0.47, *p-value* = 0.002) with fish genome size (Fig. 2, Supplementary Table S6).

**Figure 3:**
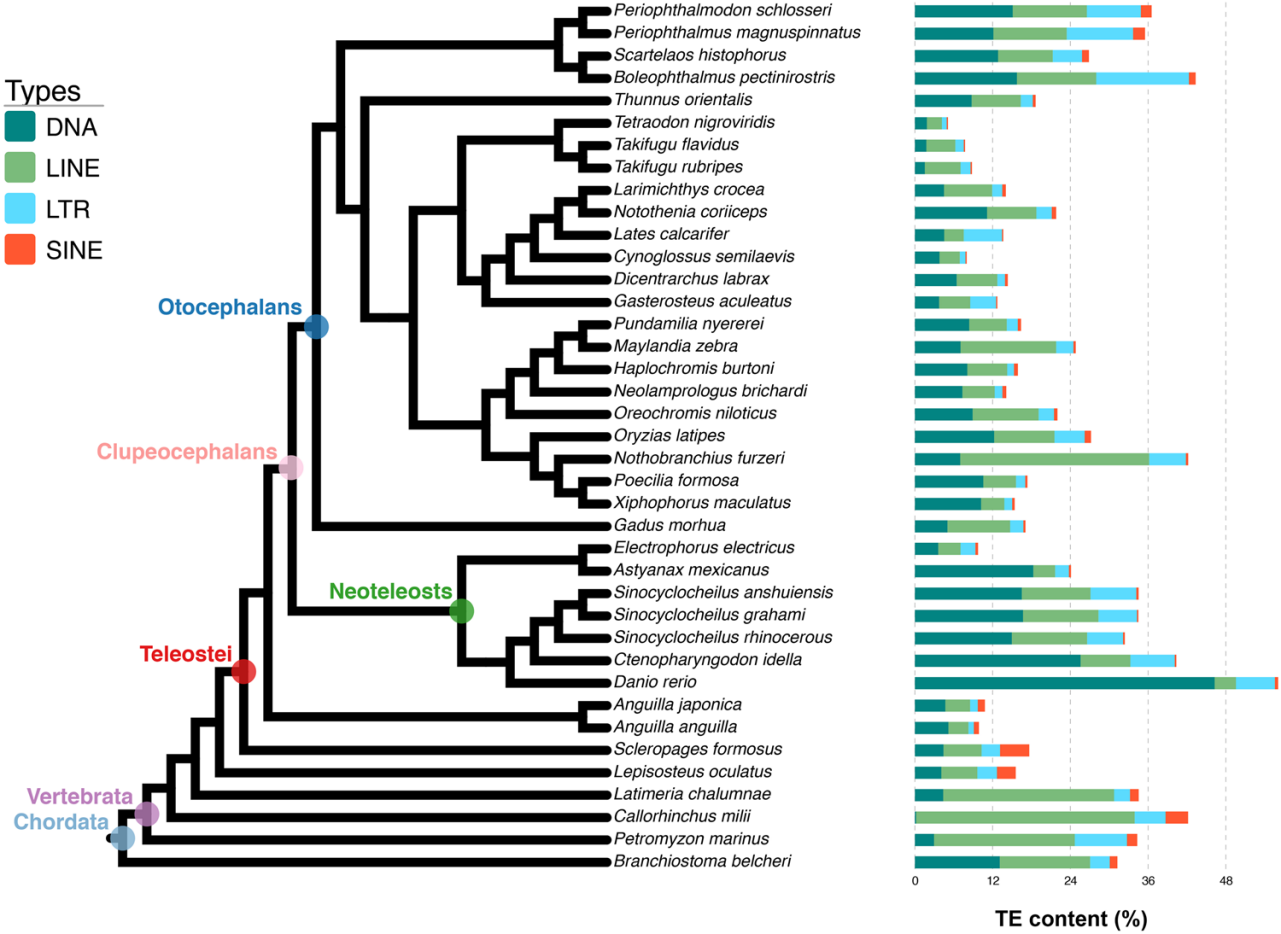
DNA transposon and retrotransposon levels in various fish genomes (Lancelet added). The percentages of DNA transposons, LTR, LINE, and SINE retrotransposons are presented.

Similar to the results of previous studies^22–24^, our data showed (Fig. 3, Supplementary Table S4) fish genomes to not always be dominated by DNA transposons, but also by LINEs in many species, as in the elephant shark (*Callorhinchus milii*), which had very few DNA transposons. In addition, most of the fish genomes studied appeared to be particularly poor in SINEs^24^. We then tested the relationship across genome size, DNA transposon, and retrotransposon (including LTR, LINE, and SINE) content; results of the analysis showed a positive correlation between retrotransposon content and genome size. This finding was statistically supported by our correlation analysis (Pearson correlation r = 0.39, *p-value* = 0.013), and LTR content was positively correlated (Pearson correlation r = 0.43, *p-value* = 0.006) with fish genome size (Supplementary Table S6).

**Figure 4:**
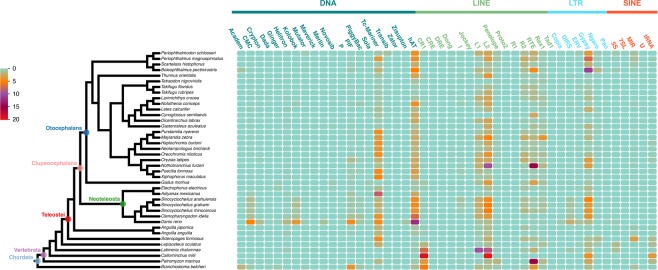
Diversity and abundance of TE superfamily in all fish genomes investigated in this study (Lancelet added). Results are presented using a heat map; content of superfamilies is shown by squares with colour gradient.

Further, to analyse TE content and distribution in fish, we calculated the levels of each TE superfamily in each species (Fig. 4, Supplementary Table S5). Our results showed *Tc/mariner*, *hAT*, *L1*, *L2*, and *Gypsy* to be widespread and the most predominant TE superfamilies in the fish genomes included in this study; distribution of other superfamilies was more erratic and species-dependent. Notably, the Cyprinidae lineage fish species (*Sinocyclocheilus anshuiensis*, *S. graham*, *S. rhinocerous*, *Ctenopharyngodon idella*, and *Danio rerio*) had the highest level of TE diversity among the species studied. Among the early diverging fishes (*C. milii*, *Latimeria chalumnae*, *Lepisosteus oculatus*, and *Petromyzon marinus*) and *Branchiostoma belcheri*, without teleost-specific whole genome duplication event, TEs of the *CR1* superfamily were predominant, although the abundance of *CR1* was very low in the fishes that diverged more recently. The levels of each TE superfamily appeared to be highly specific and species-dependent. This was particularly true for *Gypsy* in *Boleophthalmus pectinirostris*, *L2* and *RTE* in *Nothobranchius furzeri*, *Tc/mariner* in *Astyanax mexicanus, hAT* in *D. rerio, CR1, L1*, and *L2* in *L. chalumnae*, and *CR1* and *L2* in *C. milii*. We also evaluated the relationship between genome size and superfamily content. Our results showed that the higher levels of *Helitron*, *Maverick*, *Kolobok*, *CMC*, *P*, *DIRS*, *I*, *L1*, *L2*, and *5S* superfamilies positively correlated with genome size (Supplementary Table S6).

### TE transposition history and activity during fish evolution

**Figure 5:**
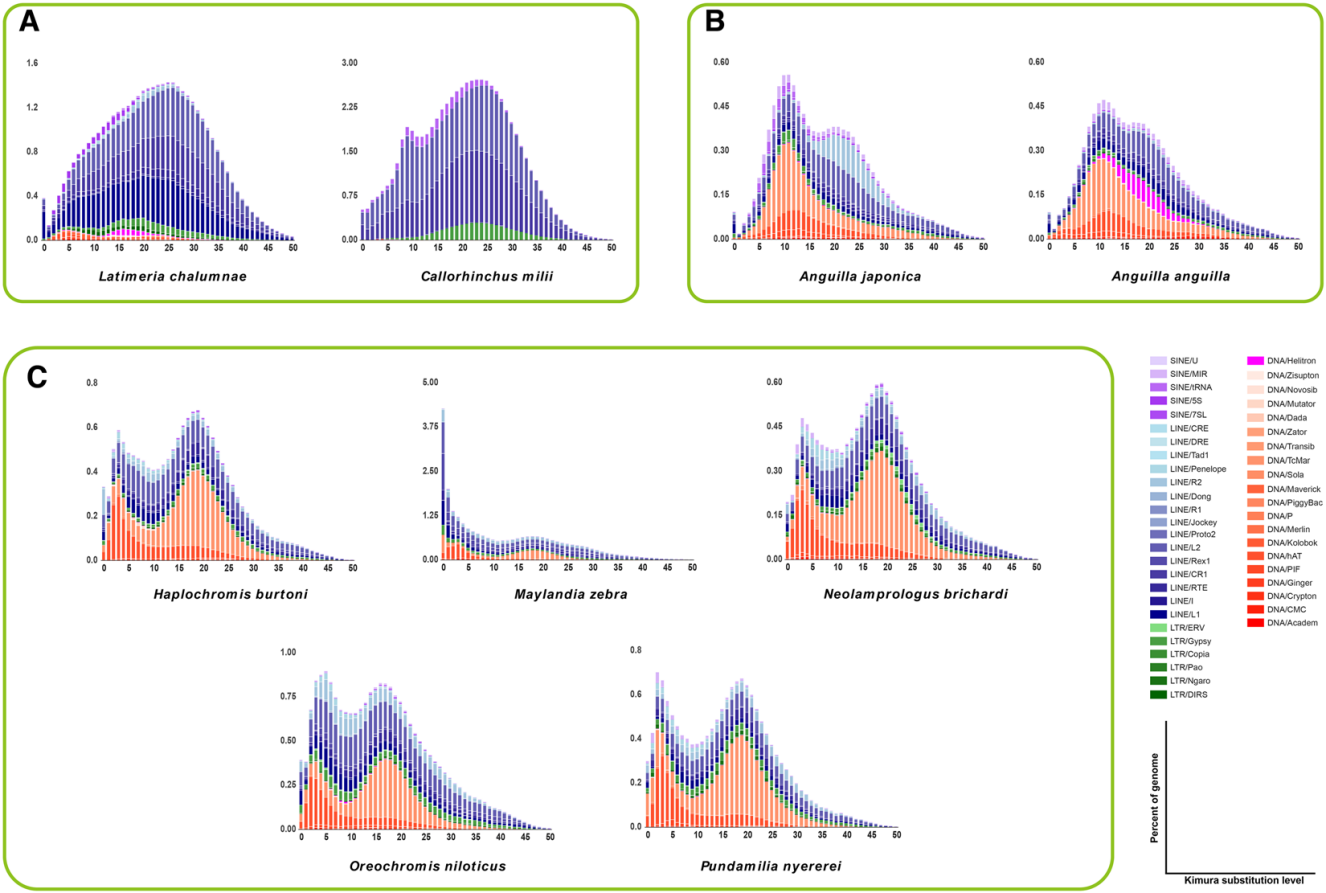
Kimura distance-based copy divergence analyses of transposable elements in coelacanth and elephant shark (**A**), eels (**B**), and Africa cichlids (**C**). The graphs represent genome coverage for each TE superfamily in the different genomes analysed. Clustering was performed according to their Kimura distances (*K-value* from 0 to 50). Copies clustering on the left side of the graph did not greatly diverge from the consensus sequence and potentially corresponded to recent events while sequences on the right side likely corresponded to older divergence.

The percentages of TE in the genome of each species were clustered based on their *K-values* (Fig. 5, Supplementary Fig. S1). Notably, copy divergence appeared to be correlated with activity age, with very similar copies (low *K-values*) being indicative of somewhat recent activity (shown on the left side of the graph) while divergent copies (high *K-values*) were likely generated by older transposition events (shown on the right side of the graph)^22^.

Indeed, each peak in the graph indicated a transposition/TE burst. Transposition bursts are common in fish, and they generally have at least one or two of them. In this process, there is usually a continuous increase in the number of active transposons before transposon “explosion”, and a continuous decline in the number of active transposons after transposon explosion. In most fish genomes, the rate at which the number of active transposons increases is smaller than the rate at which the number of active transposons declines; therefore, most of the fish genomes contain fewer ancient copies (*K-values* > 25) than recent copies (*K-values* < 25). However, we observed an opposite trend in *C. milii* and *L. chalumnae* (Fig. 5A). Interestingly, there were also some notable superfamily-dependent differences, occurring even between closely related species with similar TE landscapes. For example, in Japanese and European eels there were obvious differences in *R2* and *Helitron* transposon bursts, respectively (Fig. 5B). In African cichlids, which generally have two transposon bursts across all TE superfamilies, we observed a recent burst in the *Maylandia zebra* (Fig. 5C).

## Discussion

Transposable elements are important evolutionary components of the genome. Although various studies have been published addressing TE function and diversity^24,25^, the distribution and role of TEs in some species, especially fish, remain largely unknown owing to their complicated genomes^24^. In this study, we used species-specific TE libraries in FishTEDB, along with that of additional nine species, to analyse different classifications of TEs. Abundance, diversity, activity, and evolution of TE were explored and related to genome size and evolutionary history of fish.

With over 33,900 known species (FishBase, http://www.fishbase.org/, version 02/2018), fish comprise the majority of vertebrates. It is, therefore, not surprising that remarkable differences in morphology, population structure, and genome size have been observed across fish species. Differences in genome size, in particular, can be up to 379.5 times (0.35–133 Gb)^16^. In this study, we observed a considerable variation in TE content (5–56%) across the fish species analysed. Such variation was not only restricted to the overall levels; according to our statistical analysis of the different classifications, diversity may also be more variable and complex. Fish genomes predominantly contained DNA transposons and LINEs, whereas SINEs were the least abundant. Focusing on TE superfamilies, *Tc/mariner*, *hAT*, *L1*, *L2*, and *Gypsy* were found to be the most widespread among the fish genomes analysed. Most TEs show patchy distribution, indicating multiple events of loss and gain. However, there were some exceptions to this trend in TE diversity. In the elephant shark (Chondrichthyes), for example, the most prevalent TEs were the LINE superfamilies *L2* and *CR1*, rather than *Tc/mariner* and *hAT*. SINEs were also well represented, whereas only a few DNA transposons were detected. Therefore, our results also hinted that the TE landscapes in cartilaginous fish might be more similar to that of jawless fish rather than of bony fish^26^. In the African coelacanth (Sarcopterygii) genome, *CR1, L1*, and *L2* (LINEs) were predominant. However, in this species, the DNA transposons appeared to have recently undergone transposition, and SINEs were not well represented. These features also existed in some tetrapods, for example, Squamata, Testudines, Crocodilia, and Aves^24^. The data, therefore, indicate that TE landscape in African coelacanth might be similar to that of tetrapods. This is consistent with previous studies on the phylogenetic relationships of these species^27^. In conclusion, fish TEs were regularly distributed, and the relationship across species with similar distribution regularity was consistent with the phylogenetic relationship. This indicated that TEs play a vital role in fish evolution.

Our analysis of TE superfamilies suggested a critical role of the *CR1* superfamily in vertebrate evolution. In fact, among the earliest-diverging fishes (*C. milii*, *L. chalumnae*, *L. oculatus*, and *P. marinus*), *B. belcheri*, and tetrapods (terrestrial animals), *CR1* elements appeared to have a strong genomic contribution and were often widely distributed^22,24^. However, teleost contains fewer of these elements, hence suggesting that the *CR1* superfamily existed in ancestral vertebrates, and a significant loss occurred during the evolution of fish. Nevertheless, these elements were preserved, and proliferated from aquatic to terrestrial transition in tetrapods. Although previous studies indicated that TEs are important to genome evolution and could influence piscine adaptation to various habitats^28^, additional studies would be necessary to uncover the full function and evolutionary role of *CR1* superfamily in fish and other species.

With the exception of elephant sharks and African coelacanths, the presence and levels of some superfamilies appeared to be highly species-specific. For example, we observed *Gypsy* in *B. pectinirostris*, *L2* and *RTE* in *N. furzeri*, *Tc/mariner* in *A. mexicanus*, and *hAT* in *D. rerio*. In fact, the losses and gains of specific TEs during evolution appeared to primarily determine the content and distribution of different superfamilies in each species. Because genome defense machinery (e.g., DNA methylation, Piwi-interacting small RNAs) regulates TEs, the loss-and-gain process must be associated with host genomes^29^. Previous studies had indicated that TEs could have biological significance owing to their interaction with the host genome, akin to how species interact with ecosystems. The similarity between genomes and ecosystems was first drawn in 1989 by Holmquist^30^, who suggested that genome components may be different niches. These niches include the darkly stained, heterochromatic bands of chromosomes and TEs. Like organisms in their habitats, TEs proliferate and use resources inside the genome environment while interacting with each other^31^. Similarly, the *Red Queen* paradigm applies to interactions between host genomes and TEs, describing an antagonistic relationship that is continually evolving. Researchers have proposed these analogies to be useful in understanding TE abundance and diversity^32^. Combined with the theory of natural selection, TEs that compete with the host genome (harmful TEs) are more likely to be eliminated, whereas TEs beneficial to the host genome are more likely to be conserved. Therefore, superfamilies that are highly specific in some fish species should be considered important players in genome evolution and may be related to the biological characteristics of the species itself.

Like gene number and intron number, TE content is also a crucial genomic parameter. Many studies have described a positive relationship between TE levels and genome size^23,33–35^, and TEs have been universally recognised as a driver of genome size. Our analysis supports this conclusion in fish. Through further correlation analysis, we also confirmed that the effect of retrotransposons (Class I) on genome size was higher than that of DNA transposons (Class II). Of the various types of retrotransposons, LTRs appeared to be significantly correlated with genome size. However, despite the various DNA transposons (*Helitron*, *Maverick*, *Kolobok*, *CMC*, and *P*), LTRs (*DIRS*), LINEs (*L1*, *L2*, and *I*), and SINEs (*5S*) being positively correlated with genome size, whether these TEs drive genome size remains unclear, since most of them were present only at low levels in the fish genomes. Thus, while understanding the full function of TEs would require further study, there was indeed a general trend in fish, whereby the TE content increased with increase of genome size.

In addition to analysing the relationship between TE content and genome size, we also evaluated TE evolution and activity concerning transposition bursts. Transposition bursts occur at least once or twice, if not more, over the evolutionary history of a fish. In fact, there are active and inactive periods of TE throughout the TE ‘lifecycle’, which begins with the invasion of TE into a new genome (via a horizontal transfer event) or the evolution of a new, distinct TE lineage from a previously existing one (via a genetic mutation). Although the new element can establish itself into the genome, the host can also mount a defence against this change and proliferation can be curtailed. However, if the insertion is in some way beneficial to the host, then the TE will be conserved, and co-evolution of the element and the host will occur^36–39^. Thus, transposition bursts are likely to be associated with significant evolutionary events, as is supported by previous studies that linked speciation with a high TE activity^5,40,41^. In the present study, the content of *M. zebra* transposition burst was the highest across all the species studied. We also observed an unusually high proportion of recent bursts in the *M. zebra* of African cichlids. African cichlids are famous for their large, diverse, and replicated adaptive radiations in the Great Lakes of East Africa^42^. The activity of TE is closely related to species formation and adaptive radiation^41,43,44^. Therefore, based on our data, we believe that TEs may have the potential for continued differentiation. However, the phenomenon of TE burst is not unique; *Lates calcarifer* appears to have undergone a similar process. However, we could not speculate whether this phenomenon is related to adaptive radiation, since the existence of adaptive radiation in the evolutionary process of *L. calcarifer* has not yet been reported. Additionally, we could not rule out other factors such as environmental adaptation^45–47^. In Japanese and European eels, there are obvious differences in the *R2* and *Helitron* transposon bursts, respectively, despite their similar TE landscapes. This may have occurred during or after differentiation of their common ancestors, and preserved henceforth.

## Conclusions

In this report, we present an overview of TE abundance, diversity, activity, and evolution in fish with varying genome sizes and positions in the fish tree of life. High levels of diversity and patchy distribution were the main characteristics of TEs in the fish genomes analysed. In combination with ‘genomic ecology’ and TE ‘lifecycle’ theory, our data suggested that differential TE bursts may have actively contributed to essential evolutionary events. The *CR1* TE superfamily also appeared to play an important role in the differentiation of aquatic and terrestrial animals. Although further studies would be required to explore the relationship between TE burst/activity and vertebrate evolution, this study provides significant insight into the role of TE activity, specificity, and diversity in fish evolution and genome size, and highlights the application of FishTEDB.

## Methods

### Data collection and species-specific TE library construction

All genomes used in this study were downloaded from public databases (Supplementary Table S1). We directly used the zebrafish TE library in Repbase (http://www.girinst.org/repbase/). TE libraries of 30 species were downloaded from FishTEDB (http://www.fishtedb.org/) (Supplementary Table S1). Other TE libraries were generated using *de-novo*, homology-based, and structure-based methods. *De-novo* identification of TEs was performed using RepeatModeler (http://www.repeatmasker.org/RepeatModeler/, version 1.0.7). For the structure-based method, we used MGEScan-non-LTR^48^, LTR_STRUC^49^, MGEScan-LTR (http://darwin.informatics.indiana.edu/cgi-bin/evolution/daphnia_ltr.pl), and TESeeker^50^. REPCLASS (https://github.com/feschottelab/REPCLASS, version 1.0) and TEclass^51^ were used to classify TEs. A more detailed pipeline had been described in our previous study^21^.

### TE annotation and statistical analysis

RepeatMasker version 4.0.5 (http://www.repeatmasker.org/RMDownload.html) was used to mask the genomes. Notably, the “-a” and “-lib” default parameters were applied. Pearson correlation analysis via “cor.test ()” function in R language was applied to analyse the correlation between genome size and TE content.

### Phylogenetic tree construction

Since genome analysis of the species used in this study had already been conducted, most of their phylogenetic relationships are clear. Therefore, the phylogenetic tree was constructed by combining NCBI Taxonomy (https://www.ncbi.nlm.nih.gov/taxonomy/?term=) with existing literature^52–58^.

### TE divergence distribution

To estimate TE “age” and transposition history in fish, we performed a copy-divergence analysis of the TE superfamilies, based on their Kimura 2-parameter distances (*K-values*)^59^. Kimura distances between genome copies and TE consensus from the library were determined using buildSummary.pl, calcDivergenceFromAlign.pl, and createRepeatLandscape.pl (in RepeatMasker util directory) on alignment files (.align files) after genome masking. Transition and transversion rates were calculated for these alignments, and then transformed to Kimura distances^59^ with the following equation: *K* = −1/2 ln(1 − 2*p* − *q*) − 1/4 ln(1 − 2*q*), where *q* is the proportion of sites with transversions, and *p* is the proportion of sites with transitions.

## Supplementary information

Supplementary information: Evolution and diversity of transposable elements in fish genomes

Supplementary Table S1, Table S2, Table S3, Table S4, Table S5, Table S6
