# Supplementary material for: Evolution and diversity of transposable elements in fish genomes
Source: Sci Rep. 2019 Oct 28;9:15399. doi: 10.1038/s41598-019-51888-1 (PMC6817897; doi:10.1038/s41598-019-51888-1)
Supplement: Supplementary file 1 — Supplementary information: Evolution and diversity of transposable elements in fish genomes [file 41598_2019_51888_MOESM1_ESM.pdf]

Evolution and diversity of transposable elements in fish genomes

Feng Shao [1], Minjin Han [2] and Zuogang Peng [1*]

[1] Key Laboratory of Freshwater Fish Reproduction and Development (Ministry of Education), Southwest University School of Life Sciences, Chongqing 400715, China

[2] State Key Laboratory of Silkworm Genome Biology, Key Laboratory for Sericulture Functional Genomics and Biotechnology of Agricultural Ministry, Southwest University, Chongqing 400715, China

*Corresponding author: pzg@swu.edu.cn

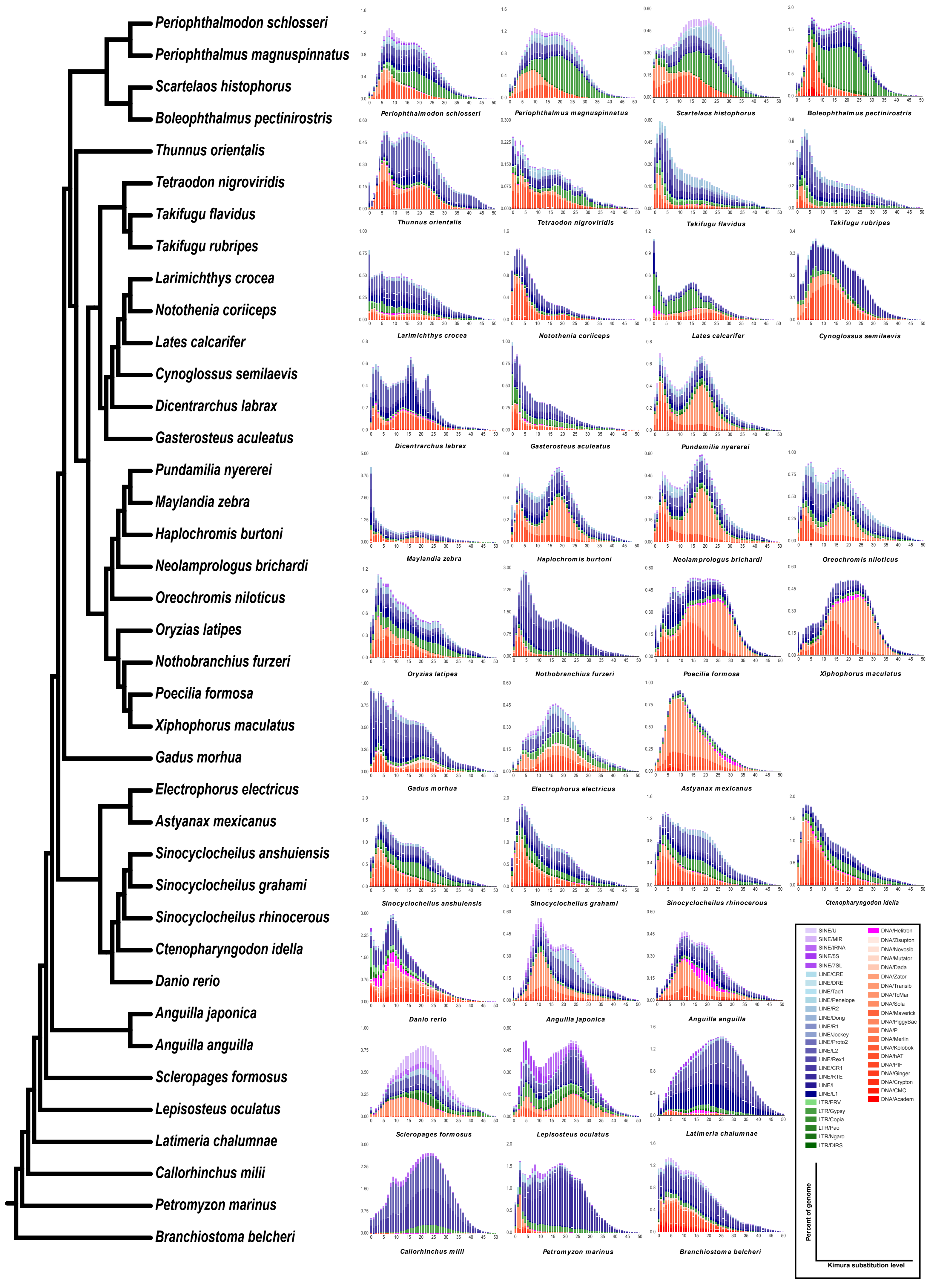

Figure S1: Kimura distance-based copy divergence analysis of TEs in fish genomes (Lancelet added)
